# Position statement of the Brazilian Academy of Rhinology on the use of antihistamines, antileukotrienes, and oral corticosteroids in the treatment of inflammatory sinonasal diseases^☆^

**DOI:** 10.1016/j.bjorl.2017.01.002

**Published:** 2017-01-21

**Authors:** Olavo de Godoy Mion, João Ferreira de Mello, Daniel Lorena Dutra, Nilvano Alves de Andrade, Washington Luiz de Cerqueira Almeida, Wilma Teresinha Anselmo-Lima, Leonardo Lopes Balsalobre Filho, Jair de Carvalho e Castro, Roberto Eustáquio dos Santos Guimarães, Marcus Miranda Lessa, Sérgio Fabrício Maniglia, Roberto Campos Meireles, Márcio Nakanishi, Shirley Shizue Nagata Pignatari, Renato Roithmann, Fabrizio Ricci Romano, Rodrigo de Paula Santos, Marco César Jorge dos Santos, Edwin Tamashiro

**Affiliations:** a Universidade de São Paulo (USP), Disciplina de Otorrinolaringologia, São Paulo, SP, Brazil; b Universidade de São Paulo (USP), Faculdade de Medicina, São Paulo, SP, Brazil; c Universidade de São Paulo (USP), Faculdade de Medicina, Departamento de Otorrinolaringologia, São Paulo, SP, Brazil; d Universidade de São Paulo (USP), Faculdade de Medicina de Ribeirão Preto, Departamento de Otorrinolaringologia, Ribeirão Preto, SP, Brazil; e Universidade Federal de São Paulo (UNIFESP), Ciências da Saúde, São Paulo, SP, Brazil; f Universidade Federal de São Paulo (UNIFESP), Otorrinolaringologia, São Paulo, SP, Brazil; g Universidade Federal de Minas Gerais (UFMG), Faculdade de Medicina, Belo Horizonte, MG, Brazil; h Universidade de São Paulo (USP), Faculdade de Medicina de Ribeirão Preto, Ribeirão Preto, SP, Brazil; i Universidade Federal da Bahia (UFBA), Faculdade de Medicina, Disciplina de Otorrinolaringologia, Salvador, BA, Brazil; j Hospital Instituto Paranaense de Otorrinolaringologia, Centro de Rinite e Alergia, Curitiba, PR, Brazil; k Universidade do Estado do Rio de Janeiro (UERJ), Rio de Janeiro, RJ, Brazil; l Universidade de Brasília (UnB), Faculdade de Medicina, Brasília, DF, Brazil; m Universidade Federal de São Paulo (UNIFESP), Departamento de Otorrinolaringologia e Cabeça e Pescoço, São Paulo, SP, Brazil; n Universidade Luterana do Brasil (ULBRA), Faculdade de Medicina, Otorrinolaringologia, Canoas, RS, Brazil; o Mount Sinai Hospital, Department of Othorhinolaryngology, Toronto, Canada; p Universidade de São Paulo (USP), Faculdade de Medicina, Ciências, São Paulo, SP, Brazil; q Universidade de São Paulo (USP), Faculdade de Medicina de Ribeirão Preto, Departamento de Oftalmologia, Otorrinolaringologia e Cirurgia de Cabeça e Pescoço, Ribeirão Preto, SP, Brazil

**Keywords:** Rhinitis, Rhinosinusitis, Antihistamines, Glucocorticoids, Leukotriene antagonists, Rinites, Rinossinusites, Anti-histamínicos, Glicocorticoides, Antagonistas de leucotrieno

## Abstract

**Introduction:**

Inflammatory conditions of the nose and paranasal sinuses are very prevalent in the general population, resulting in marked loss of quality of life in affected patients, as well as significant work, leisure, and social activity losses. These patients require specific and specialized treatment. A wide range of oral medications are available.

**Objective:**

The present document is aimed to clarify, for professionals treating patients with inflammatory sinonasal diseases, both specialists and general practitioners, specific oral therapies in noninfectious nasal inflammatory conditions.

**Methods:**

The methodology used to create this article included the search for the key words: oral corticosteroids, antihistamines, antileukotrienes, rhinitis, rhinosinusitis in the MEDLINE and EMBASE databases in the last 5 years. Since no relevant article was found for the text on the subject of interest in the last 5 years, the search was extended for another 5 years, and so on, according to the authors’ needs.

**Results:**

Relevant literature was found regarding the use of antihistamines, antileukotrienes and oral corticosteroids in these conditions. The Brazilian Academy of Rhinology emphasizes, after extensive discussion by the collegiate, key points in the treatment with these drugs.

**Conclusion:**

There is support in the literature for the use of these drugs; however, final considerations about the role of each of them have been made.

## Resumo

**Introdução:**

As afecções inflamatórias do nariz e seios paranasais são muito prevalentes na população geral, causam acentuada perda de qualidade de vida dos pacientes afetados, gerando perdas significativas das atividades de trabalho, lazer e sociais. Estes pacientes necessitam de tratamento específico e especializado e uma ampla gama de medicações orais estão disponíveis.

**Objetivo:**

O presente documento tem por objetivo esclarecer àqueles que tratam das doenças nasossinusais inflamatórias, tanto especialistas quanto generalistas, sobre as terapêuticas orais nas afecções inflamatórias nasais não infecciosas.

**Método:**

A metodologia utilizada para elaboração deste artigo incluiu a busca das palavras chave: corticosteroides orais, anti-histamínicos, antileucotrienos, rinite, rinossinusite nos bancos de dados MEDLINE e EMBASE nos últimos 5 anos. Não sendo encontrado artigo relevante para o texto sobre o assunto de interesse nos últimos 5 anos, a busca poi extendida por mais 5 anos, e assim por diante, de acordo com a necessidade dos autores.

**Resultados:**

Literatura relevante foi encontrada com relação ao uso dos anti-histamínicos, antileucotrienos e corticosteroides orais nestas afecções. A Academia Brasileira de Rinologia resalta, após amplo debate do colegiado, pontos-chave no tratamento com estes medicamentos.

**Conclusão:**

Há respaldo na literatura para o uso destes medicamentos, entretanto considerações finais acerca do papel de cada deles foram feitas.

## Introduction

Inflammatory conditions of the nose and paranasal sinuses are the most prevalent group of diseases in the general population. These diseases, such as allergic and non-allergic rhinitis, acute and chronic rhinosinusitis, with and without nasal polyposis, result in a marked decrease in the quality of life of affected patients, generally causing significant work, leisure, and social activity losses. These patients require specific and specialized treatment.

Oral medications are extremely important in the treatment of inflammatory diseases of the nose and paranasal sinuses, as well as in the treatment of infectious diseases of the upper airways. Although some classes of drugs have been used for decades, new molecules have recently been made available.

Due to the prevalence of these diseases, there are very high direct and indirect costs associated with treatment, especially in the long term. The cost associated with treatment should not be ignored, and the correct use of these drugs can result in lower costs for both the patients and their families, as well as for public health and society.

The aim of this document is to clarify for professionals who treat inflammatory sinonasal diseases, both specialists and general practitioners, nasal oral therapies for these noninfectious diseases. Through a review of the scientific evidence, the Brazilian Academy of Rhinology provides a practical and up-to-date view of the most frequently used nasal oral medications, except for medications that have antimicrobial agents in their formulation.

The methodology used to create this article included the search for the key words: oral corticosteroids, antihistamines, antileukotrienes, rhinitis, and rhinosinusitis in the MEDLINE and EMBASE databases in the last 5 years. No article was found relevant to the text on the subject of interest in the last 5 years and, therefore, the search was extended to another 5 years, and so on, according to the authors’ needs.

## The role of histamine and leukotrienes in nasal inflammatory diseases

### Histamine

Histamine has an important physiological role and can bind to 4 different receptors^1^ (Table 1). Through these bindings, it acts on immunoregulation and allergic inflammation. In allergic rhinitis, the histamine released into the nasal mucosa binds to H1 receptors and triggers vasodilation, increased vascular permeability, pruritus, increased glandular secretion, and nerve-ending stimulation.

**Table 1:** Antihistamines and histamine receptors.

Receptors	G Protein	Main activity
H1	Gαq	Atopy – Gell and Coombs Type I Reaction
H2	Gαs	Digestive tract
H3	Gαi	Central nervous system
H4	Gαi	Chemotaxis of eosinophils and mast cells

Adapted from the III Consensus on rhinitis.^1^

Histamine receptors are classified as G-protein receptors in active or inactive form. Histamine stabilizes its active structuring, while antihistamines, acting as inverse agonists, stabilize the inactive conformation.^2^

### Leukotrienes

**Figure 1:**
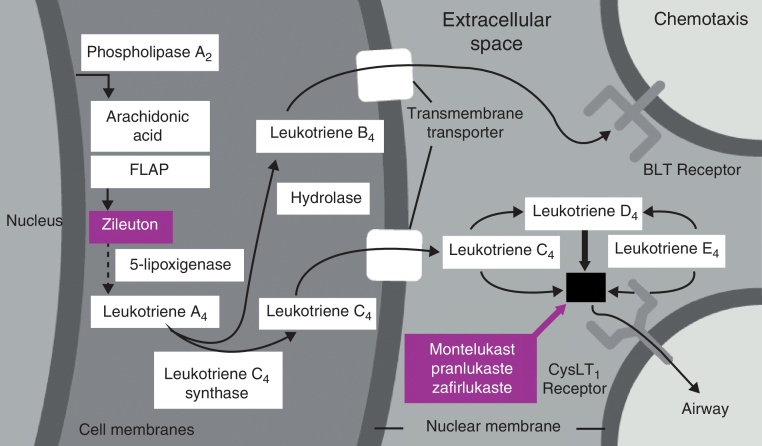
Eicosanoid pathway leading to leukotriene formation.

Evidence of the role of leukotrienes in disease pathophysiology comes from studies of the immediate and late phases in allergen-triggering. That did not happen after contact with methacholine.^3^, ^4^ Analysis of the secretion of patients with persistent rhinitis showed high levels of C4 and D4 cysteine leukotrienes^5^ and LTC4.^6^ Due to the intense capacity of leukotrienes to cause inflammation, which is thousands of times greater than that of histamine, it has been speculated that nasal obstruction and congestion are directly associated with this class of mediators^7^ (Fig. 1).

Nasal polyposis is a chronic inflammatory disease of the upper respiratory tract that affects 2–4% of the population and 2/3 of patients with acetylsalicylic acid-sensitive asthma. The histology of polyps is similar to that of asthma, with abundant eosinophils, mast cells, and high levels of proinflammatory cystenyl leukotrienes.^8^

It has been proposed that one of the potential causes of chronic rhinosinusitis with nasal polyps is the presence of defects in the eicosanoid pathway, more strongly associated with acetylsalicylic acid intolerance.^9^ Specifically, the increased synthesis of pro-inflammatory leukotrienes and the decreased synthesis of anti-inflammatory prostaglandins have been the accepted mechanism, not only for chronic rhinosinusitis with nasal polyps in acetylsalicylic acid-sensitive patients but also in those tolerant to this drug.^10^

Regarding leukotrienes and chronic rhinosinusitis, there are many data about their action on inflammation reduction, especially concerning eosinophils and eicosanoid pathway.^11^ Montelukast showed a reduction in eosinophilic inflammation, cytokine viability and production in nasal polyps.^12^ It has been demonstrated that calcium (Ca^+^) influx into mast cells through the activation of Ca^+^ channels release stimulates the production of C4 leukotrienes, which in turn activates a higher Ca^+^ influx.^13^

## Antihistamines

Antihistamines are considered the gold standard medication for the treatment of allergic rhinitis.^2^

They decrease the allergic inflammatory reaction through their action on H1 receptors, by interfering with the action of histamine on sensory neurons and small vessels. The kappa-beta nuclear transcription factor inhibition also reduces the antigenic presentation, the expression of cytokines and cell adhesion molecules. They also reduce mast cell activation in a dose-dependent manner.^11^

H1 antihistamines are classified into two groups. The first-generation drugs rapidly cross the blood–brain barrier and occupy H1 receptors located on the postsynaptic membrane of histaminergic neurons. Most of these antihistamines were marketed before pharmacological studies were required by regulatory agencies thus, pharmacokinetic and pharmacodynamic data are not available for most of them.^11^

On the other hand, second-generation drugs create a lower potential for sedation (Table 2) and pharmacokinetic and pharmacodynamic data have been published for several groups, such as healthy adults, the elderly, children, patients with renal failure, etc. (Table 3). Similarly, their interaction with food and other drugs is known. It is emphasized that after intake discontinuation, the histamine response suppression in the allergic tests lasts from 1 to 5 days.^11^

**Table 2:** Second-generation antihistamines and effects on the central nervous system.

Drug	Doses mg	Sedation observed in studies on rhinitis or urticaria, alone
Bilastine	20	1.8–5.8%
Cetirizine	10	6–8.5%
Desloratadine	5	1.1–3.7%
Ebastine	10	1.4–2.7%
	20	<2–3%
Fexofenadine	120	<2–3%
	180	1.7–4.5%
Levocetirizine	5	0.7–6.7%
Loratadine	10	2.2–6.6%
Rupatadine	10	2.7–10%

Adapted from Simons and Simons.^2^

**Table 3:** Pharmacokinetics and pharmacodynamics of some antihistamines in adults.^11^

	Generation	*T*_max_ (h)	Half-life (h)	Start of action (h)	Duration of effect (h)
Chlorpheniramine	1st	2.8	27.9	3	24
Diphenhydramine	1st	1.7	9.2	2	12
Hydroxyzine	1st	2.1	20.0	2	24
Bilastine	2nd	1.2	14.5	2	24
Cetirizine	2nd	1.0	6.5	0.7	≥24
Desloratadine	2nd	1–3	27	2–2.6	≥24
Fexofenadine	2nd	1–3	11	1–3	24
Levocetirizine	2nd	0.8	7	0.7	>24
Loratadine	2nd	1.2	7.8	2	24
Rupatadine	2nd	0.75	6	2	24

Adapted from Simons and Simons.^2^

The potential adverse effects of first-generation antihistamines can be divided according to their action on other receptors, as shown in Table 4.

**Table 4:** Potential adverse effects of first-generation antihistamines.^2^

Action site	Effect
H1 receptor in central nervous system	Sedation and decreased attention, cognition, learning, memory and psychomotor performance
Muscarinic receptor	Dry mouth and eyes, urinary retention, sinus tachycardia, mydriasis, and constipation
Serotonin receptor	Increased appetite and weight gain
Alpha-adrenergic receptor	Dizziness and postural hypotension
Cardiac ion channels	Increased QT interval and ventricular arrhythmia

Adapted from Simons and Simons.^2^

There is strong scientific evidence for their action in improving allergic rhinitis and allergic rhinoconjunctivitis symptoms.^2^, ^11^, ^14^ Second-generation antihistamines prevent and improve symptoms such as sneezing, nasal pruritus, and rhinorrhea, which characterize the immediate response of the Gell and Coombs Type I allergic reaction classification. However, they show a diminished effect on nasal congestion (late phase) (Table 5). They also control ocular symptoms such as erythema, tearing, pruritus and edema.^2^, ^11^, ^14^

**Table 5:** Effect of medications on allergic rhinitis symptoms.

	Sneezing	Rhinorrhea	Nasal obstruction	Nasal pruritus
Oral antihistamines	**++**	**++**	+	**+++**
Oral decongestants			**+++**	
Antileukotrienes	**+**	**+**	**+**	**+**

Modified from the III Consensus on Rhinitis and American Academy of Otorhinolaryngology and Head and Neck Surgery.^1^

According to the American Academy of Otorhinolaryngology and Head and Neck Surgery and the Allergic Rhinitis and Its Impact on Asthma consensus, it is recommended to use second-generation oral antihistamines for patients with allergic rhinitis. Although they are not as effective as intranasal corticosteroids, antihistamines possess the advantage of cost, rapid action onset, and maintenance of efficiency with regular use in mild and moderate cases. Their greatest benefit comes with regular use; however, their administration “when necessary” is of great usefulness as temporary rescue medication.^2^, ^14^

The first and second-generation antihistamine doses for adults and children are shown in Table 6.

**Table 6:** Antihistamine presentation and doses.

Anti H1	Presentation	Doses	
		Children 2–12 years	12 years
*Classic (first generation)*			
Clemastine	Syrup: 5 mg/mL Tablet: 1 mg	<1 year: 2.5 mL 12/12 h 1–3 years: 2.5–5 mL 12/12 h 3–6 years: 5 mL 12/12 h 6–12 years: 7.5 mL 12/12 h	20 mL 12/12 h 1 tablet 12/12 h
Dexchlorpheniramine	Syrup: 25 mg/mL Tablet: 2 mg Pill: 6 mg	6 years: 1.2 mL 8/8 h 6–12 years: 2.5 mL 8/8 h	5 mL or 1 tablet 8/8 h Maximum of 12 mg/day
Hydroxyzine	Syrup or tablet	Up to 6 years: 50 mg/day >6 years: up to 100 mg/day	Up to 150 mg/day
Promethazine	Syrup: 5 mg/5 mL Tablet: 25 mg	1 mg/kg/day 2–3 times/day	20–60 mg/day
Cyproheptadine	Elixir: 2 mg/5 mL Tablet: 4 mg	2–6 years: 2 mg 8/8 h Maximum 8 mg/day 6–12 years: 4 mg 8/8 h Maximum 16 mg/day	4 mg 8/8 h Maximum 16 mg/day

*Non-classic (second generation)*			
Loratadine	Oral solution: 5 mg/5 mL Tablet: 10 mg	>2 years: <30 kg: 5 mg/day >30 kg: 10 mg/day	10 mg/day
Cetirizine	Drops: 10 mg/mL Tablet: 10 mg	2–6 years: 2.5 mg/dose 12/12 h 6–12 years: 5 mg/dose 12/12 h	10 mg/day
Rupatadine	Tablet: 10 mg	–	10 mg/day
Epinastine	Syrup: 10 mg/5 mL Tablet: 10 mg Tablet: 20 mg	6–12 years: 5–10 mg/day	20 mg/day
Levocetirizine	Drops: 5 mg/mL Tablet: 5 mg	6 years: 1.25 mg/dose 12/12 h 6–12 years: 5 mg/day	5 mg/day
Desloratadine	Oral solution: 2.5 mg/5 mL Tablet: 5 mg	2–5 years: 1.25 mg/d 6–11 years: 2.5 mg/day	5 mg/day
Ebastine	Syrup: 1 mg/mL Tablet: 10 mg	2–6 years: 2.5 mg/day 6–12 years: 5 mg/day	10 mg/day
Fexofenadine	Oral solution: 6 mg/mL Capsules: 60 mg Tablet: 120 mg	6 months–2 years: 15 mg/dose 12/12 h 2–11 years: 30 mg/dose 12/12 h	60 mg 12/12 h 120 mg/d
Bilastine	Tablet: 20 mg	–	20 mg/day

Modified from Mion.^15^

Antihistamines are not recommended in the treatment of acute bacterial rhinosinusitis,^16^ but they can be used for relieving sneezing and rhinorrhea symptoms in viral cases.^8^ In patients with chronic rhinosinusitis, with or without polyposis, there is no recommendation for the use of oral antihistamines, except in allergic patients. According to evidence-based medical studies, there is improvement in clinical and endoscopic scores in these patients.^8^

The intake of first-generation antihistamines may occasionally cause intense dizziness, confusion, delirium, coma, and respiratory depression. In children, on the other hand, there may be paradoxical effects, such as excitement, irritability, hyperactivity, insomnia, and hallucination.^2^

In contrast, the second-generation drugs, at the usual doses, are practically free of adverse effects on the central nervous system and action on the muscarinic, serotonin and alpha-adrenergic receptors.^11^ Their safety in special populations is described in Table 7.

**Table 7:** Adverse effects in special populations.^11^

	1st generation	2nd generation
Renal/liver failure	Few studies. It may be potentially associated with adverse effects.	Data evaluated for each drug. The drug package leaflet should be consulted for possible dose changes.
Elderly	Impairs cognition, memory and attention. It can lead to falls, delirium, and incontinence.	Data evaluated for each drug. The drug package leaflet should be consulted for more information.
Pregnant women	Diphenhydramine and chlorpheniramine are classified as Class B^a^ drugs (FDA). Irritability and drowsiness have been reported in infants.	Cetirizine and loratadine are classified as Class B^a^ drugs (FDA). Desloratadine, fexofenadine and levocetirizine are Class C^a^ drugs (FDA). No adverse effects have been reported in infants.
Neonates	May cause irritability, drowsiness and respiratory depression.	No effect on central nervous system.
Children	Potential risk of adverse effects.	Long-term safety for cetirizine, desloratadine, fexofenadine, levocetirizine and loratadine has been demonstrated.

Adapted from Simons and Simons.^2^

a Risk classification of drug use in pregnancy, according to the Food and Drug Administration (FDA). Category A – adequate and well-controlled studies have not shown any risk to the fetus in the first trimester of pregnancy (there is no evidence of risk in other trimesters); B – studies on animal reproduction have not shown a risk to the fetus and there are no adequate and well-controlled studies in pregnant women; C – animal reproduction studies have demonstrated adverse effects on the fetus and there are no adequate and well-controlled studies in humans; however, the potential benefits may justify the drug use in pregnant women despite the potential risks; D – there is evidence of risk to the fetus based on adverse reactions from investigational studies or post-marketing studies; however, the potential benefits may justify the drug use in pregnant women despite the potential risks; X – animal or human studies have demonstrated fetal alterations or evidence of risk to the human fetus based on adverse reactions from investigational or post-marketing studies and the risks involved in the drug use in pregnant women do not justify the potential benefits. FDA Pregnancy Categories. Available at: http://www.drugs.com/pregnancy-categories.html [accessed 02.04.16].

### Decongestants associated with antihistamines

Nasal decongestants are subdivided into oral and topical formulations. As they are alpha-adrenergic agonists, their main effect is vasoconstriction.^1^ Pseudoephedrine is the most commonly used decongestant, in combination with antihistamines (Table 8, Table 9). This combination has a better effect than each drug alone in controlling nasal symptoms, but the chance of adverse effects such as insomnia, headache, dry mouth, and irritability increases.^17^, ^18^ Their use reduces hyperemia, edema and nasal congestion^18^ and their safety is known in single daily doses of up to 240 mg for the control of nasal obstruction in seasonal allergic rhinitis, other respiratory allergies and rhinosinusitis.^18^

**Table 8:** Association of first-generation antihistamines with decongestants.^1^

Association	Presentation	Doses for children	Doses for adults and children older than 12 years
Azatadine + pseudoephedrine	Pill 1 mg azatadine + 120 mg pseudoephedrine Syrup 0.5 mg azatadine + 30 mg pseudoephedrine/mL	>6 years: 5 mL every 12 h 1–6 years: 2.5 mL every 12 h	1 tablet every 12 h 10–20 mL every 12 h
Brompheniramine + phenylephrine^a^	Syrup 5 mL with 2 mg brompheniramine + 5 mg phenylephrine Drops 1 mL with 2 mg brompheniramine + 2.5 mg phenylephrine Tablet: 12 mg brompheniramine + 15 mg phenylephrine	>2 years: 2.5–5 mL every 6 h >2 years: 2 drops per kg divided every 8 h	15 –30 mL every 6 h 1 tablet every 12 h
Brompheniramine + pseudoephedrine	Syrup 1 mL with 0.2 mg brompheniramine + 3 mg pseudoephedrine Capsules with 4 mg brompheniramine + 60 mg pseudoephedrine	>6 months: 0.25–0.30 mL/kg/dose every 6 h	20 mL every 6 h 1 capsule every 6 h
Triprolidine + pseudoephedrine	Syrup: every 5 mL 1.25 triprolidine + 30 mg pseudoephedrine Tablet: 2.5 mg triprolidine + 60 mg pseudoephedrine	2–5 years: 2.5 mL every 6 h 6–12 years: 5 mL every 6 h	10 mL every 6 h 1 tablet every 6 h

Adapted from III Consensus on rhinitis.^1^

a No evidence of clinical effect on nasal obstruction.

**Table 9:** Association of second-generation antihistamines with oral decongestants.^1^

Association	Presentation	Doses for children	Doses for adults and children older than 12 years
Fexofenadine + pseudoephedrine	Tablet 60 mg + 120 mg pseudoephedrine		1 tablet every 12 h
Loratadine + pseudoephedrine	Tablet 5 mg loratadine + 120 mg pseudoephedrine	Weight > 30 kg: 5 mL every 12 h Weight < 30 kg: 2.5 mL every 12 h	1 tablet every 12 h
	Tablet 24 h 10 mg loratadine + 240 mg pseudoephedrine Syrup 1 mg loratadine + 12 mg pseudoephedrine/mL		1 tablet/day
Ebastine + pseudoephedrine	Capsules 10 mg ebastine + 120 mg pseudoephedrine		1 tablet every 12 h
Desloratadine + pseudoephedrine	Capsules 2.5 mg + pseudoephedrine		1 tablet every12 h
Cetirizine + pseudoephedrine	Capsules 5 mg + 120 mg pseudoephedrine		1 tablet every 12 h

A recent study carried out by the Food and Drug Administration (FDA) to evaluate the efficacy of phenylephrine in controlling nasal obstruction has shown that it has a similar effect to placebo when given at a dose of up to 40 mg every 4 h.^19^

There is no evidence for the efficacy of decongestants in cases of acute bacterial rhinosinusitis in children or adults, as well as in patients with chronic rhinosinusitis (with or without polyposis).^20^, ^21^

Pseudoephedrine has minimal hepatic metabolism and is eliminated, unaltered, in the urine. Its half-life is 4–8 h.^21^, ^22^ Oral decongestants should be prescribed with caution to the elderly, children, patients with a history of cardiac arrhythmia, angina pectoris, cerebrovascular disease, hypertension, urinary retention, hyperthyroidism and should be avoided in patients with prostatic hypertrophy and athletes, since it is considered doping.^23^, ^24^

### Antihistamines associated with antileukotrienes

An antihistamine–antileukotriene association has been recently launched in the market. Its objective is to improve the clinical effect of the drugs, either by association or potentiation of effect. Additionally, it can improve adherence to treatment by offering two different classes of drugs in a single tablet.

Some studies, mostly directed at rhinitis, have shown that montelukast has been associated with a variety of second-generation antihistamines, such as loratadine,^25^, ^26^ fexofenadine,^27^ desloratadine^28^ and cetirizine.^29^ A meta-analysis has shown that their clinical effect is superior to that of placebo.^30^ Other studies have shown that the combination is superior to that of antihistamines or antileukotrienes alone in controlling allergic rhinitis symptoms.^29^, ^31^

Few studies have evaluated the combination of levocetirizine and montelukast, but the results have been promising, superior to the associations with other antihistamines. The combination of levocetirizine and montelukast showed superior results, with a beneficial additive effect in the treatment of persistent allergic rhinitis.^32^, ^33^ There are studies showing that such a combination brings benefits in preventing symptoms in patients with poor response to monotherapy and in controlling symptoms, especially nocturnal ones.^34^, ^35^

## Leukotriene antagonists

The leukotriene receptor antagonist, montelukast, is used in the control of allergic diseases, such as asthma and rhinitis, as it is a receptor blocker that binds with high affinity and selectivity to the cysteine receptors found in the airways.^36^, ^37^ It has no bronchodilator effect, but it improves lung function of patients with moderate and severe asthma,^38^ In addition to improving the symptoms of rhinitis,^39^ sleep apnea,^40^ and conjunctivitis,^41^ and may be used as adjunctive therapy in chronic urticaria.^42^ The GINA (Global Initiative for Asthma),^43^ PRACTALL (Practicing Allergology)^44^ and ARIA (Allergic rhinitis and its impact on asthma)^11^ guidelines recommend the use of montelukast as a therapeutic agent for the control of asthma and rhinitis.

### Affinity and selectivity for the cysteine receptor

When montelukast binds with high affinity and selectivity to cysteine receptors (CysLT), it promotes the physiological blocking of leukotrienes C1 to C4, D and E. This binding does not occur with other respiratory receptors (cholinergic, prostanoid, beta-adrenergic). Montelukast, as well as another leukotriene antagonist, zafirlukast, are also potent ligands of the CysLT receptor, more strongly than pranlukaste and other equivalent compounds (LM-1507 and LM-1484).^45^

### Safety and side effects

Montelukast has shown to be a drug with a high safety profile and is recommended for the treatment of asthma and rhinitis by consensus and global guidelines.^11^, ^44^, ^45^ The overall incidence of adverse events is considered low (Table 10). The Cochrane Foundation rated the drug as being safer than long-acting beta-2 agonists.^46^

**Table 10:** Side effects of antileukotrienes.^48^, ^49^, ^50^, ^51^, ^52^, ^53^, ^54^, ^55^, ^56^, ^57^, ^58^, ^59^

Adverse effects of montelukast		
Overall effects	Pharyngitis, fever, infection	Comparable to placebo^47^
Effects on lower airways	Worsening of asthma	Comparable to placebo^47^
Effects on central nervous system	Irritability, aggressiveness, hallucinations Suicide?	Related to other drugs used together? Symptom improvement after treatment discontinuation^48^, ^49^, ^50^, ^51^, ^52^
Vascular system	Churg-Strauss Syndrome? (Vasculitis) Angioedema	Not fully clarified^53^, ^54^
Skin	Urticaria	^53^
Hepatic	Hepatitis	53, 55
High doses of up to 1000 g	Malaise, vomiting, abdominal pain and hyperactivity	No serious accounts in relation to overdose^56^, ^57^

### Efficacy of montelukast in allergic rhinitis

Several studies since the 1990s have investigated the possible efficacy of leukotriene antagonists in the treatment of allergic rhinitis.^58^, ^59^

The antagonist zafirlukast was evaluated in allergic rhinitis, which showed some protection,^59^ as well as pranlukaste.^60^ The use of montelukast resulted in greater efficacy, including good cost–benefit,^61^ although it is less effective than nasal corticosteroids.^35^

Several authors have evaluated the action of montelukast in studies with more than 1000 patients with seasonal^62^, ^63^ and persistent rhinitis,^64^ confirming the improvement of all cardinal symptoms of allergic rhinitis, with effect on sleep and quality of sleep, ocular symptoms, allergic rhinoconjunctivitis and quality of life in general.^60^, ^62^, ^63^, ^64^

The efficacy of antileukotrienes in allergic rhinitis and asthma, after more than 15 years of use, has been widely demonstrated. Montelukast has been very well evaluated for the treatment of seasonal and perennial allergic rhinitis. It results in significant improvements in nasal and ocular symptoms between 1 and 3 days, as well as in nocturnal symptoms, sleep quality and quality of life.^60^

### Antileukotrienes and chronic rhinosinusitis with and without nasal polyps

Nasal polyposis is a chronic inflammatory disease of the upper respiratory tract affecting 2–4% of the population and 2/3 of asthmatic patients with acetylsalicylic acid sensitivity.^8^

Although the pathophysiology of chronic rhinosinusitis suggests the use of antileukotrienes, double-blind randomized clinical trials do not support theoretical studies regarding the efficacy of leukotriene inhibitors so clearly.^65^

Leukotriene antagonists, such as montelukast, zafirlukast and zileuton were evaluated in studies involving patients with chronic rhinosinusitis with nasal polyposis and Aspirin-Exacerbated Respiratory Disease (AERD).^66^, ^67^ The results were not clear. Many uncontrolled open studies have suggested the benefits of antileukotrienes in symptomatology, nasal polyp size and tomographic scores.^68^ Other results include significant improvement in headache, pain and facial pressure, auditory discomfort, dental pain, purulent nasal discharge, post-nasal drip, nasal congestion and obstruction, as well as olfactory symptoms.^69^ These studies conclude that leukotriene-modifying drugs, if added to standard medications, including corticosteroids, result in improved nasal symptoms in patients with chronic rhinosinusitis with and without polyposis.^70^, ^71^, ^72^

However, data from double-blind, randomized controlled studies do not consistently support the benefit of antileukotriene therapy in patients with chronic rhinosinusitis.^66^, ^73^ Although antileukotrienes are effective in patients with AERD, they are no more effective than in acetylsalicylic acid-tolerant patients.^74^, ^75^

Regarding the association of montelukast with intranasal corticosteroids, there are studies that demonstrate the efficacy of their combined use in chronic rhinosinusitis. Montelukast added to intranasal corticosteroids improves symptoms in patients with chronic rhinosinusitis, with an excellent safety profile.^76^

For these reasons, the action of antileukotrienes, when analyzed from the point of view of evidence-based Medicine, discloses a limited level of efficacy and has a low degree of recommendation in patients with chronic rhinosinusitis with nasal polyposis.^77^

Montelukast was, to date, the most commonly used antileukotriene. Its anti-inflammatory action, especially those related to the eosinophil and its cytokines has been demonstrated by several studies. Another important factor when considering montelukast is its high safety and tolerability, even in children.^11^ What is clearly understood is its usefulness in allergic patients with asthma and patients with acetylsalicylic acid intolerance. These are the patients with chronic rhinosinusitis who should receive antileukotrienes therapy, either as an adjunctive therapy or not, in the postoperative and maintenance periods.

## Oral corticosteroids

Glucocorticosteroids (GCs) are a class of drugs with effects on several cell functions, and their important effect on the mechanisms involved in inflammation make them one of the main modalities of treatment in autoimmune and inflammatory diseases, such as asthma, allergy, rheumatoid arthritis, multiple sclerosis, and inflammatory bowel diseases.^78^ This characteristic also provides a relevant role in nasal inflammatory diseases. However, their therapeutic benefits are limited by the side effects associated with their prolonged use and high doses.

These effects include osteoporosis, skin atrophy, diabetes, glaucoma, cataracts, hypertension, avascular necrosis, infection and increased abdominal fat.^79^ Table 11 shows the main systemic GCs used in clinical practice with their equivalence table and anti-inflammatory potency.^80^ We use the terminology “corticosteroid” (CS) as synonymous with GC.

**Table 11:** Equivalence, anti-inflammatory potency and half-life of corticosteroids.

	Approximate equivalence dose in mg	Relative anti-inflammatory potency^a^	Biological half-life (h)
Hydrocortisone	20	1	8–12
Prednisone	5	3.5–4.0	12–36
Prednisolone	5	4.0	12–36
Methylprednisolone	4	5.0	12–36
Dexamethasone	0.75	30	36–72
Betamethasone	0.6	30	36–72
Deflazacort	7.5	2.5–3.5	24–36

Adapted from hormonal anti-inflammatory drugs: glucocorticoids.^80^

a In comparison to hydrocortisone (cortisol).

Corticosteroids act on protein synthesis. When they penetrate the cells, they bind to receptors called glucocorticosteroid receptors and go into the cell nucleus, where they trigger genomic effects. They have two action mechanisms: the first is called transactivation, when it induces the synthesis of proteins such as lipocortin-1, beta-adrenergic receptors, secretory leukoprotease inhibitor. They also have a transrepression action, where there is inhibition of synthesis of inflammatory cytokines and adhesion molecules, among others. Such mechanism seems to be the most relevant in inflammatory diseases, and also the least related to the adverse effects of this class of drugs.^1^, ^81^

### Acute bacterial rhinosinusitis

A Cochrane review has reported that there is no robust current evidence for the use of systemic corticosteroids as monotherapy for the treatment of acute rhinosinusitis.^82^

In patients who received an association of corticosteroids and systemic antibiotics, there seems to be some benefit such as symptomatic relief, although current data are limited. The short-term benefit occurs with the reduction in pain scores, and there seems to be improvement in the more acute symptoms of facial pain and headache.^82^, ^83^ There is no evidence of better long-term results (over 2 weeks) and after 10 days for the improvement observed in the groups treated with the association of corticosteroids and antibiotics resembles those observed in the placebo groups.^83^, ^84^ Therefore, it appears corticosteroids can bring symptomatic relief in the short term as adjunctive therapy.

### Chronic rhinosinusitis

A recent systematic review suggests an important role for systemic corticosteroids in treating exacerbations of chronic rhinosinusitis (CRS) with polyps, being indicated for a short, intermittent treatment (1–3 weeks).^85^ This study cites 3 previous systematic reviews for use in CRS,^86^, ^87^, ^88^ demonstrating an association with symptom improvement, quality of life questionnaires, and polyp score when compared to placebo, in a total of 5 randomized controlled trials. However, the trials showed improvement only in the short-term, for approximately 2–3 weeks, with limited follow-up of 2–6 months.

In CRS without polyps, the evidence in the literature is more limited, the studies are heterogeneous and lack a control group, without randomized controlled trials, demonstrating a lower level of evidence. The use of systemic corticosteroids in patients with CRS without polyps requires studies with a more robust methodology.

A meta-analysis and a systematic review evaluated the role of corticosteroids in endoscopic functional surgery of the paranasal sinuses.^89^ Eighteen studies were included, for a total of 1309 patients. Studies with mixed populations of CRS with and without polyps and the use of systemic and/or topical corticosteroids were evaluated. The results indicated a significant intraoperative benefit: significant reduction of blood loss, reduced surgical time and quality of the surgical field improvement. There was no significant difference regarding postoperative pain and postoperative symptom scores. However, the postoperative endoscopic evaluation scores were significantly better in the corticosteroid group. The subgroup of patients with CRS with polyps had a lower rate of recurrence when compared to controls.

### Allergic rhinitis

The use of corticosteroids for a short period of time may be a therapeutic option in allergic rhinitis patients who are not responsive to other treatments.^90^ The existence of other very effective treatment options, together with steroid use potential adverse effects, especially for a prolonged period of time, does not justify their systematic and routine use in allergic rhinitis. Therefore, they are not considered as a first-line treatment.^91^

## Final considerations

The use of second-generation antihistamines is recommended over the first-generation ones due to improved safety profile. Regarding drowsiness or sedation, the dose of second-generation drugs should be considered, as well as time of use and individual sensitivity of each patient.

The use of oral decongestants is useful in relieving symptoms of acute nasal obstruction. Caution is advised regarding their use due to their potential side effects.

The use of antihistamines associated with antileukotrienes becomes important in the presence of monotherapy failure.

Leukotriene receptor antagonists can be used in adults and children with seasonal allergic rhinitis and in preschool children with persistent allergic rhinitis due to their efficacy, high safety, and tolerability.^16^ This line of medications can still be used as an adjuvant treatment in the treatment of chronic rhinosinusitis.

Oral corticosteroids are useful as rescue medication for chronic rhinosinusitis with polyps, and are usually prescribed for a short period of 1–3 weeks. In chronic rhinosinusitis without polyps, the evidence for corticosteroid use is very limited. Therefore, the analysis of a possible benefit against the potential risks of using oral corticosteroids should guide the clinical decision-making. Similarly, in acute bacterial rhinosinusitis, the physician should individually evaluate each patient, determine symptom severity and the risks of oral corticosteroid use and consider their use as an option for symptomatic relief.

## Conflicts of interest

The authors declare no conflicts of interest.
